# Efficient hybrid fuzzy weighted 3D FCNN with TSO PSO optimization for accurate multi modal MRI brain tumor classification

**DOI:** 10.1038/s41598-025-19889-5

**Published:** 2025-10-15

**Authors:** Liu Feng, Guo Hao, Wang Jian

**Affiliations:** Shanxi Medical University, Fenyang College, Fenyang, 032200 Shanxi People’s Republic of China

**Keywords:** Brain tumor segmentation, Computer aided diagnosis, Fuzzy logic systems, Transit search algorithm, Convolutional neural networks, Cancer, Health care, Medical research, Engineering

## Abstract

Detecting and segmenting brain tumors from 3D MRI images is a challenging and time-intensive task for clinicians. This research introduces an innovative hybrid architecture for deep learning, comprising a 3D fully convolutional neural network (3D-FCNN), an interval type-2 fuzzy weighting system, and a hybrid transit search optimization-particle swarm optimization (Hybrid-TSO-PSO) algorithm. The proposed models, 3D-FCNN-Hybrid-TSO-PSO and 3D-FCNN-SVM, employ type-2 fuzzy weighting to diminish the quantity of trainable parameters and expedite training on MRI volumetric data. The Hybrid-TSO-PSO optimization approach integrates the heuristic strengths of TSO with the rapid convergence attributes of PSO, enhancing learning stability and augmenting the precision of segmentation and classification. Assessments were conducted on the BraTS 2019, BraTS 2020, and a portion of the BraTS 2021 datasets, comprising 300 3D MRI images (230 high-grade HGG and 70 low-grade LGG glioma specimens). During the testing phase, the 3D-FCNN-Hybrid-TSO-PSO model attained an accuracy of 98.1%, sensitivity of 98.9%, specificity of 95.0%, and a Dice score of 0.987, whereas the 3D-FCNN-SVM model earned an accuracy of 95.2%. This method not only enhances accuracy but also decreases training duration by as much as sixfold relative to traditional architectures, serving as an efficient and precise diagnostic aid for the identification and classification of brain cancers.

## Introduction

Brain tumors, both primary and secondary, are among the most aggressive and life-threatening conditions of the central nervous system^1,2^. Their accelerated proliferation can impair essential neurological functions, resulting in significant disability or mortality if not identified and addressed promptly. The prognosis is heavily contingent upon the promptness and precision of the diagnosis^3^. Magnetic Resonance Imaging (MRI) is the gold standard for brain tumor evaluation due to its exceptional soft-tissue contrast and capacity to acquire various modalities namely T1-weighted, T2-weighted^4^, contrast-enhanced T1 (T1ce), and FLAIR sequences that provide complementary anatomical and pathological insights^5^. Nonetheless, the manual interpretation of MRI scans is a labor-intensive procedure that necessitates considerable competence, especially when tumor margins are indistinct or the imaging features exhibit substantial variability^6^. The escalating volume of patient data in contemporary healthcare imposes an increasing effort on radiologists, hence amplifying the danger of errors due to weariness^7^. This has generated significant interest in automated computer-aided diagnostic (CAD) systems that can aid doctors by providing consistent, precise, and rapid tumor detection and segmentation^8^.

Despite extensive research over several decades, the complete automation of brain tumor identification and segmentation continues to pose significant challenges^9^. The variability in acquisition techniques, disparities in contrast and noise levels, and the inherent uniqueness of tumor forms, sizes, and textures complicate the development of algorithms that generalize effectively across datasets and patient populations^10^. Tumor locations may exhibit analogous intensity patterns to healthy tissues, whereas imaging artifacts might further confuse the delineation of lesions. Conventional image processing methods such as intensity thresholding, edge detection, or unsupervised clustering frequently prove inadequate in complex situations, especially when utilized on multi-modal 3D MRI data^11^. The emergence of deep learning, particularly Convolutional Neural Networks (CNNs), transformed medical image analysis by facilitating end-to-end feature extraction straight from raw data^12^. However, when utilized for volumetric data, CNNs encounter specific limitations: the extensive quantity of learnable parameters heightens the likelihood of overfitting, prolongs training duration, and necessitates substantial computational resources^13^. These challenges are exacerbated when various MRI modalities are integrated for enhanced feature representation.

Recent research in the fields of medical imaging and deep learning has shown diverse applications in medical data analysis. Classification of anesthesia stages using near-infrared spectroscopy signals^14^ and improvement of deep learning algorithms for image sentiment classification^15^ have inspired the development of computational methods for analyzing MRI brain tumor images. Also, the study of craniectomy surgery in intracerebral hemorrhage^16^ emphasizes the importance of accurate imaging in brain diagnostics, which is aligned with the goal of improving brain tumor diagnosis. Innovation networks and urban structures of the medical device industry^17,18^ also indirectly contribute to the advancement of imaging technologies such as MRI, which was used in this study to classify brain tumors. In recent years, hybrid frameworks that amalgamate deep learning with fuzzy logic systems and metaheuristic optimization algorithms have garnered attention. Fuzzy logic provides a systematic approach to manage uncertainty and imprecision in medical data, facilitating adaptive feature weighting according to domain-specific regulations^19,20^. Takagi–Sugeno (T–S) fuzzy models have been employed to augment the adaptability of CNN filters, resulting in enhanced classification performance. Metaheuristic algorithms, like Transit Search Optimization (TSO) and Particle Swarm Optimization (PSO), have demonstrated efficacy in optimizing network hyperparameters and circumventing suboptimal local minima. Nevertheless, the majority of previous studies have utilized these strategies in isolation or with minimal integration, frequently resulting in either sluggish convergence or inadequate global exploration. Furthermore, numerous current models are designed for 2D slices or a singular MRI modality, hence limiting their practical utility.

**Fig. 1:**
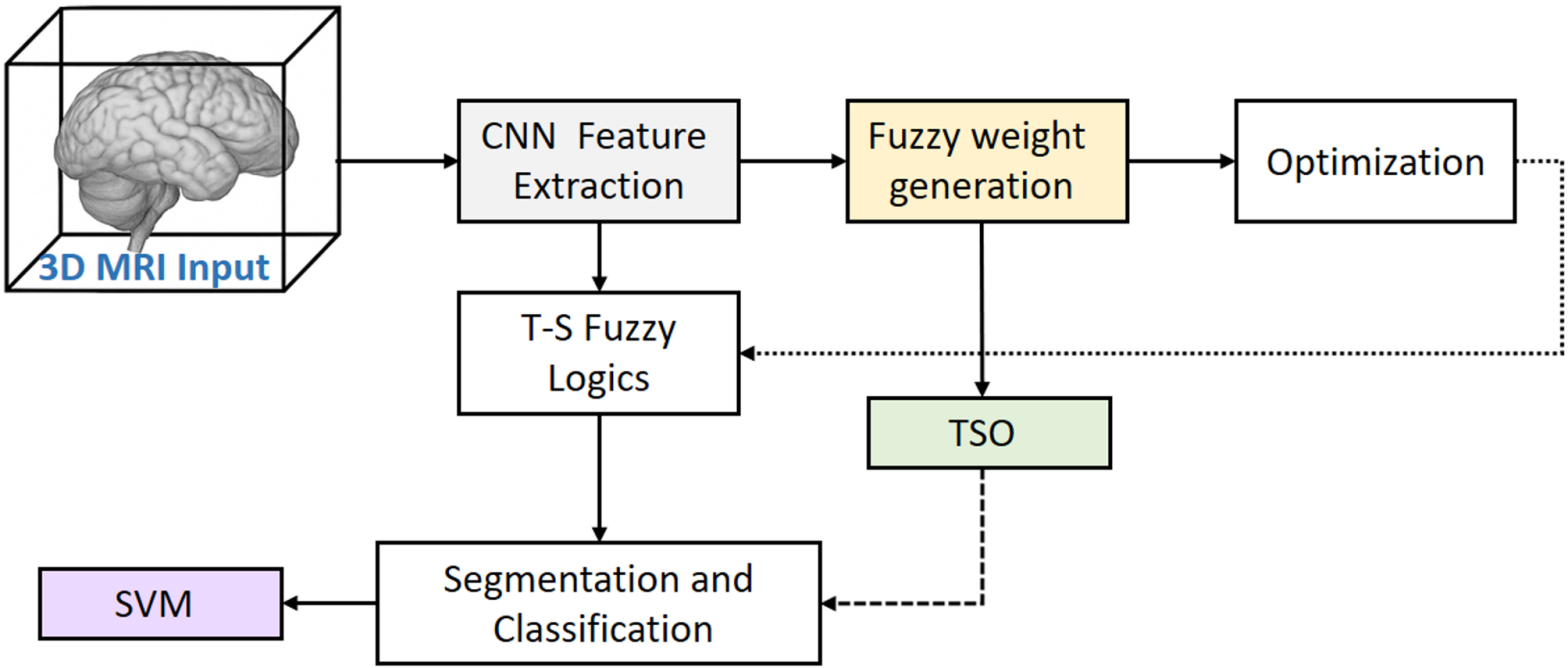
Architectural diagram of the proposed Hybrid 3D-FCNN–T-S Fuzzy–TSO/SVM approach for processing 3D MRI images.

Figure 1 shows an integrated overview of the proposed pipeline for brain tumor detection and classification based on 3D MRI images. The process starts with the input of multimodal 3D MRI images, which can include T1, T2, FLAIR, and CE-T1W sequences. In the first step, a 3D convolutional neural network (3D-FCNN) is employed as a feature extraction section to automatically identify complex spatial-volume patterns from the images. The output of this section is fed into the Takagi-Sugeno fuzzy logic module (T-S Fuzzy), which is responsible for generating the initial weights of the convolution filters. This step reduces the number of trainable parameters of the network and reduces the computational complexity by utilizing fuzzy weight generation. Then, the Transient Search Algorithm (TSO) is used to optimize the generated parameters to increase the accuracy and learning speed. In the final stage, the optimized features are directed to two classification paths: the first path uses a Fully Connected layer with a SoftMax function to separate classes, and the second path uses a Support Vector Machine (SVM) classifier to increase accuracy on feature-rich datasets.

This study presents an innovative automated framework for brain tumor classification and segmentation, integrating three synergistic components: a 3D Fully Convolutional Neural Network (3D-FCNN) for volumetric feature extraction, an Interval Type-2 Fuzzy Weighting mechanism for adaptive convolutional filter weight generation, and a Hybrid Transit Search Optimization–Particle Swarm Optimization (Hybrid-TSO-PSO) algorithm for effective parameter tuning.

Despite significant progress in brain tumor detection using deep learning, most existing models suffer from limitations such as excessive trainable parameters, high computational cost for 3D MRI volumes, and instability during optimization. Many prior studies either rely on single optimization algorithms prone to local minima or lack adaptive weighting mechanisms to reduce redundancy in convolutional layers.

The novelty of this study lies in the development of a hybrid architecture that integrates a 3D Fully Convolutional Neural Network (3D-FCNN) with an Interval Type-2 Fuzzy Weighting mechanism and a Hybrid Transit Search Optimization–Particle Swarm Optimization (Hybrid-TSO–PSO) algorithm. The fuzzy weighting significantly reduces the number of trainable parameters, accelerating training without compromising accuracy, while the hybrid optimization leverages the exploration capacity of TSO and the fast convergence of PSO to achieve stable and precise learning. This joint design enables high-accuracy segmentation and classification of brain tumors from volumetric MRI data, with up to sixfold faster training compared to conventional architectures.

The primary aim of this project is to develop a CAD system capable of accurately differentiating between high-grade gliomas (HGG) and low-grade gliomas (LGG), while concurrently segmenting the tumor region with accuracy. The suggested method seeks to mitigate the two primary shortcomings of existing 3D CNN-based systems: high computational expense and restricted generalization across various MRI datasets. The system combines fuzzy logic with hybrid metaheuristic optimization to attain computational economy and robust accuracy, rendering it appropriate for use in clinical environments with limited hardware capabilities. The main contributions of this work are as follows:

- Development of a 3D-FCNN architecture augmented with Interval Type-2 fuzzy weighting to reduce the number of trainable parameters and enhance adaptability to multi-modal MRI input.
- Introduction of a novel Hybrid-TSO-PSO optimization strategy that balances global search and local refinement for faster convergence and improved classification performance.
- Comprehensive evaluation of the proposed system on BraTS 2019, BraTS 2020, and a subset of BraTS 2021 datasets, demonstrating superior accuracy and reduced training time compared to state-of-the-art methods.

The remainder of this paper is organized as follows. Section “Related works” reviews recent literature on brain tumor detection and segmentation, emphasizing hybrid approaches combining deep learning, fuzzy logic, and metaheuristic optimization. Section “Proposed method” details the Interval Type-2 fuzzy weighting system and the Hybrid-TSO-PSO algorithm. Section “Performance evaluation” presents the proposed 3D-FCNN-Hybrid-TSO-PSO architecture and its integration strategy. Section 5 describes the datasets, preprocessing steps, and experimental protocols. Section reports and analyzes the results, including quantitative metrics and qualitative segmentation outputs. Finally, section “Conclusion” concludes the study and outlines potential directions for future research.

## Related works

The most lethal disease in the world is a brain tumor. Untreated and undiagnosed malignancies diminish survival rates. Magnetic resonance imaging (MRI) is utilized for tumor analysis; nevertheless, the vast volume of images produced complicates and prolongs patient diagnosis, thereby jeopardizing their life. Consequently, the manual detection of early-stage brain tumors is challenging.

Consequently, a sophisticated and autonomous system is essential for the prompt and precise diagnosis of brain tumors. In^21^, researchers suggested a pre-trained EfficientNetb4 model featuring a configurable learning rate and specific callbacks for efficient tumor categorization. The suggested method enhances the quality and quantity of the publically available Br35h dataset through the application of data augmentation techniques.

The study^22^ highlights the essential requirement for precise brain tumor prediction through the creation of an automated approach that integrates the Firefly (FF) algorithm with the interval type II fuzzy methodology (IT2FLS). The proposed method enhances tumor differentiation in intricate brain tissue by employing the FF algorithm to identify potential clustering locations and the IT2FLS system for final clustering. The system exhibits versatility by analyzing various image sequences from the BRATS challenge datasets (2017, 2018, and 2020), which include differing levels of complexity.

The paper^23^ formulated an algorithm that integrates the principles of artificial bee colony and the interval type II fuzzy logic system (IT2FLS) to define tumor regions amidst intricate brain tissues. The core of any treatment protocol resides in the decisiveness of oncologists, with the algorithm proposed in this research markedly improving decision-making via technology intervention.

A high-performance classification model for MS was created in^24^. The dataset was partitioned into training, validation, and test subsets. They utilized AlexNet as the foundational model and employed transfer learning to modify AlexNet for the classification of brain scans of MS patients in their study. The enhanced MS classification methodology is superior. In^25^, the researchers derived features using a custom convolutional neural network (CNN) to acquire distinct image-level representations. The suggested CNN employed various innovative techniques, including rank-based mean fusion and multi-way data augmentation. Conversely, the relationship-aware representations were derived from a graph convolutional network (GCN).

Researchers in^26^ assess the existing comprehension of TRPM2 concerning brain malignancies and investigate the impacts of prospective pharmacological interventions targeting TRPM2, including hydrogen peroxide (H2O2), curcumin, docetaxel, selenium, paclitaxel, resveratrol, and botulinum toxin.

Deformation of the brain due to head trauma leads to traumatic brain injury (TBI). The maximum principal strain (MPS) has been utilized to assess brain deformation and forecast injury, with new findings indicating that incorporating the maximum principal strain rate (MPSR) and the product of MPS and MPSR, denoted as MPS × SR, enhances the precision of traumatic brain injury (TBI) prediction. Nonetheless, there exists some ambiguity regarding the computation of MPSR. Two methodologies have been employed: one utilizing the time derivative of MPS (MPSR1) and the other use the first eigenvalue of the strain rate tensor (MPSR2). MPSR1 and MPSR2 have been utilized in prior research to forecast TBI. To quantify the disparities between the two methodologies, researchers in^27^ evaluated them over eight in-vivo head impact datasets and one in-silico dataset, discovering that 95MPSR1 was marginally greater than 95MPSR2, while 95MPS × SR1 was, on average, 4.85% larger than 95MPS × SR2.

The study paper^28^ presents a novel, precise, and optimal system for brain tumor identification. The system encompasses tasks including preprocessing, segmentation, feature extraction, optimization, and detection. The system employs a hybrid filter for preprocessing, integrating Gaussian, mean, and median filters. Thresholding and histogram methods are utilized for image segmentation. The gray level co-occurrence matrix (GLCM) is utilized for feature extraction. The optimized convolutional neural network (CNN) approach employs whale optimization and gray wolf optimization for optimal feature selection. Brain tumor identification is conducted using a CNN classifier.

Notwithstanding the prevalent application of magnetic resonance imaging (MRI) for cerebral assessment and advancements in AI-driven diagnostic techniques, the development of a precise and effective model for tumor detection and classification from MRI images continues to pose a difficulty. In^29^, a deep convolutional neural network (CNN) architecture is presented for the automatic classification of brain pictures into four categories, alongside a U-Net-based segmentation model. They evaluated the classification model using six benchmark datasets and trained the segmentation model, facilitating a direct comparison of the influence of segmentation on tumor classification in brain MRI images.

^30^ proposes a cohesive methodology for precise brain tumor classification and segmentation through the integration of various complementing techniques. The classification of brain cancers by magnetic resonance imaging (MRI) scans is essential for the non-invasive assessment of brain tumors and offers anatomical data for precise classification. Classifying brain tumors presents numerous problems, such as the intrinsic complexity of tumor heterogeneity, discrepancies in imaging procedures, and the necessity to differentiate between benign and malignant lesions. Conventional methods frequently encounter constraints in managing the intricate patterns found in medical imaging, hence requiring the use of sophisticated computational techniques. This procedure entails the analysis of MRI data to categorize brain tumors according to diverse characteristics. The proposed model combines an attention-augmented convolutional neural network (CNN), random forest (RF), and U-Net to use the advantages of attention mechanisms, ensemble learning, and semantic segmentation techniques. The attention-enhanced CNN captures intricate features by focused attention, while the RF improves classification accuracy via ensemble learning, and the U-Net guarantees precise tumor segmentation.

Table 1 presents a comparative summary of key prior studies on brain tumor analysis, covering their proposed models, datasets, evaluation metrics, and main limitations or unique contributions. This systematic overview highlights the diversity of approaches, ranging from deep learning architectures to hybrid fuzzy–metaheuristic methods, and demonstrates the incremental improvements achieved over time.

**Table 1:** Comparative summary of related works and the proposed method.

Ref.	Model	Dataset(s)	Main evaluation metrics	Key limitations / unique contributions
^21^	Pre-trained EfficientNetb4 with adjustable learning rate and callbacks; data augmentation applied	Br35h dataset (public)	Accuracy (reported in original work), potentially other classification metrics	Improves classification efficiency via transfer learning; limited to Br35h dataset, no segmentation component
^22^	Firefly (FF) algorithm + Interval Type-2 Fuzzy Logic System (IT2FLS) for clustering	BraTS challenge datasets (2017, 2018, 2020)	Accuracy, possibly Dice, Sensitivity, Specificity (original)	Combines metaheuristic clustering with fuzzy logic; adaptable to multiple MRI sequences; may have higher computational cost
^23^	Artificial Bee Colony + IT2FLS for tumor region detection	Not explicitly specified; complex brain tissue MRI	Not specified	Enhances oncologist decision-making; limited dataset details and metrics
^24^	Transfer learning with AlexNet for MS brain scan classification	Custom MS dataset (train/val/test split)	Accuracy	Focused on MS classification, not tumor; uses AlexNet adaptation
^25^	Custom CNN + rank- based mean fusion + multi-way data augmentation + GCN for relation-aware features	Not specified	Not specified (likely Accuracy, AUC)	Novel fusion of CNN and GCN; dataset details unclear
^26^	Review and analysis of TRPM2-targeted pharmacological interventions	Not applicable (review study)	Not applicable	Summarizes therapeutic agents; not a computational model
^27^	Comparative study of MPSR1 vs. MPSR2 for TBI prediction	8 in-vivo + 1 in-silico head impact datasets	Prediction precision measures	Clarifies computational differences between strain rate definitions; not tumor-specific
^28^	Hybrid preprocessing filter + GLCM features + CNN with Whale Optimization and Gray Wolf Optimization	Not specified	Accuracy, Sensitivity, Specificity (original)	Full pipeline from preprocessing to detection; uses dual metaheuristics for feature selection
^29^	CNN for classification + U-Net for segmentation; evaluated segmentation effect on classification	Six benchmark datasets (classification); tumor MRI for segmentation	Accuracy (classification), Dice (segmentation)	Directly compares classification with and without segmentation
^30^	Attention-augmented CNN + Random Forest + U-Net	Not specified	Accuracy, Dice	Combines attention mechanisms, ensemble learning, and semantic segmentation; addresses heterogeneity and imaging variability
Proposed	3D-FCNN + Interval Type-2 Fuzzy Weighting + Hybrid- TSO-PSO optimization	BraTS 2019, BraTS 2020, subset of BraTS 2021 (multi- modal MRI, 300 cases: 230 HGG, 70 LGG)	Accuracy 98.1%, Sensitivity 98.9%, Specificity 95.0%, Dice 0.987	Reduces trainable parameters via fuzzy weighting; accelerates convergence with hybrid metaheuristic; robust multi-modal 3D classification and segmentation

## Proposed method

**Fig. 2:**
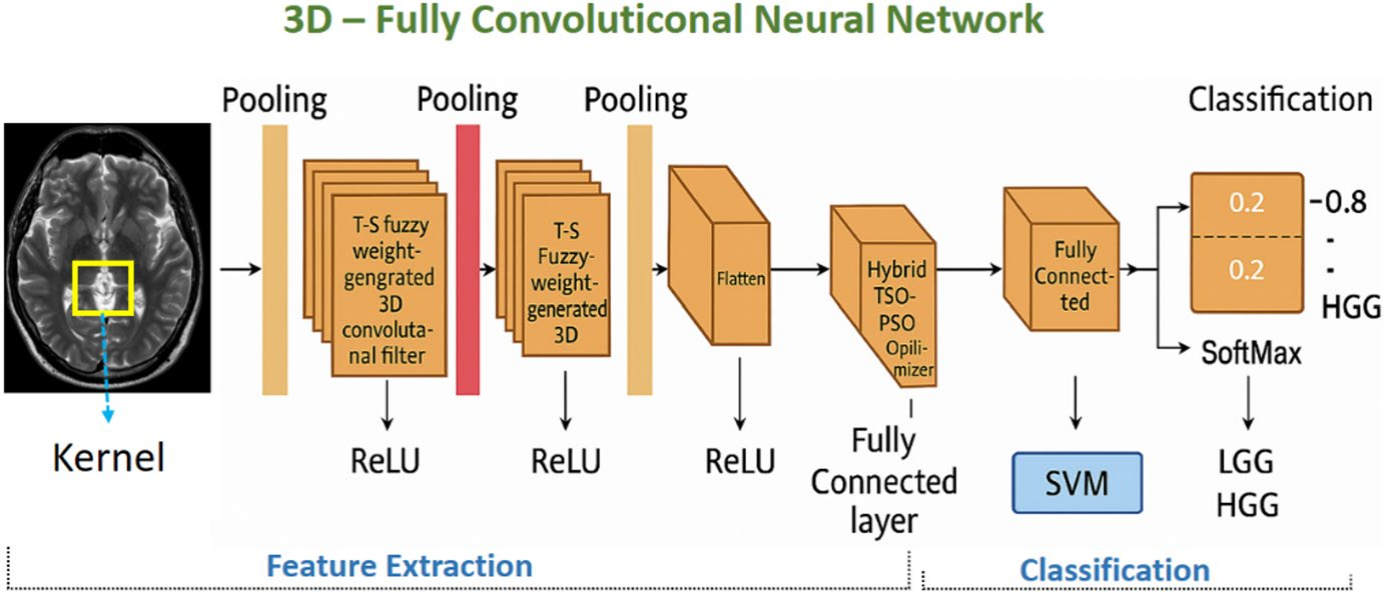
The architecture of the proposed 3D-FCNN with Hybrid-TSO-PSO approach for brain tumor classification from 3D MRI.

The proposed architecture utilizes a three-dimensional fully convolutional neural network (3D-FCNN) as the primary mechanism for automated brain tumor analysis, with its parameters refined via a hybrid metaheuristic framework integrating Transient Search Optimization (TSO) and PSO. In contrast to traditional convolutional networks that learn each filter coefficient directly, our approach employs a Takagi–Sugeno (T-S) fuzzy logic-based weight generation mechanism, significantly decreasing the number of trainable parameters while maintaining the quality of feature extraction. Figure 2 shows the complete and step-by-step architecture of the proposed 3D-FCNN model with Hybrid-TSO-PSO approach for brain tumor classification from 3D MRI images. This architecture consists of two main parts: Feature Extraction and Classification. In the feature extraction part, the input data is first processed using 3D convolutional filters whose weights are generated randomly-fuzzy by Takagi-Sugeno fuzzy logic (T-S Fuzzy Logic). Each convolution layer is combined with ReLU activation function and Pooling layer to extract multi-scale features. Next, these features are connected to the Fully Connected layer, where the Hybrid-TSO-PSO algorithm is used to optimize the parameters and weights. The classification part consists of two paths: using SVM or using Fully Connected layer with SoftMax to separate the two classes LGG and HGG.

In the feature extraction module, convolution operations are executed on the input volume, succeeded by the non-linear Rectified Linear Unit (ReLU) activation and pooling layers. Max pooling is utilized in the initial layers to preserve distinct border details, whereas average pooling is employed in subsequent stages to consolidate wider contextual information. The convolution kernels, sized at 3 × 3 × 3, are produced using the T-S fuzzy module utilizing only two descriptor parameters per filter, referred to be www and codecodecode. This substitutes the conventional method of directly learning all 27 parameters, yielding an approximate 92.6% reduction in filter-specific variables and hence diminishing computational burden in deeper layers.

**Fig. 3:**
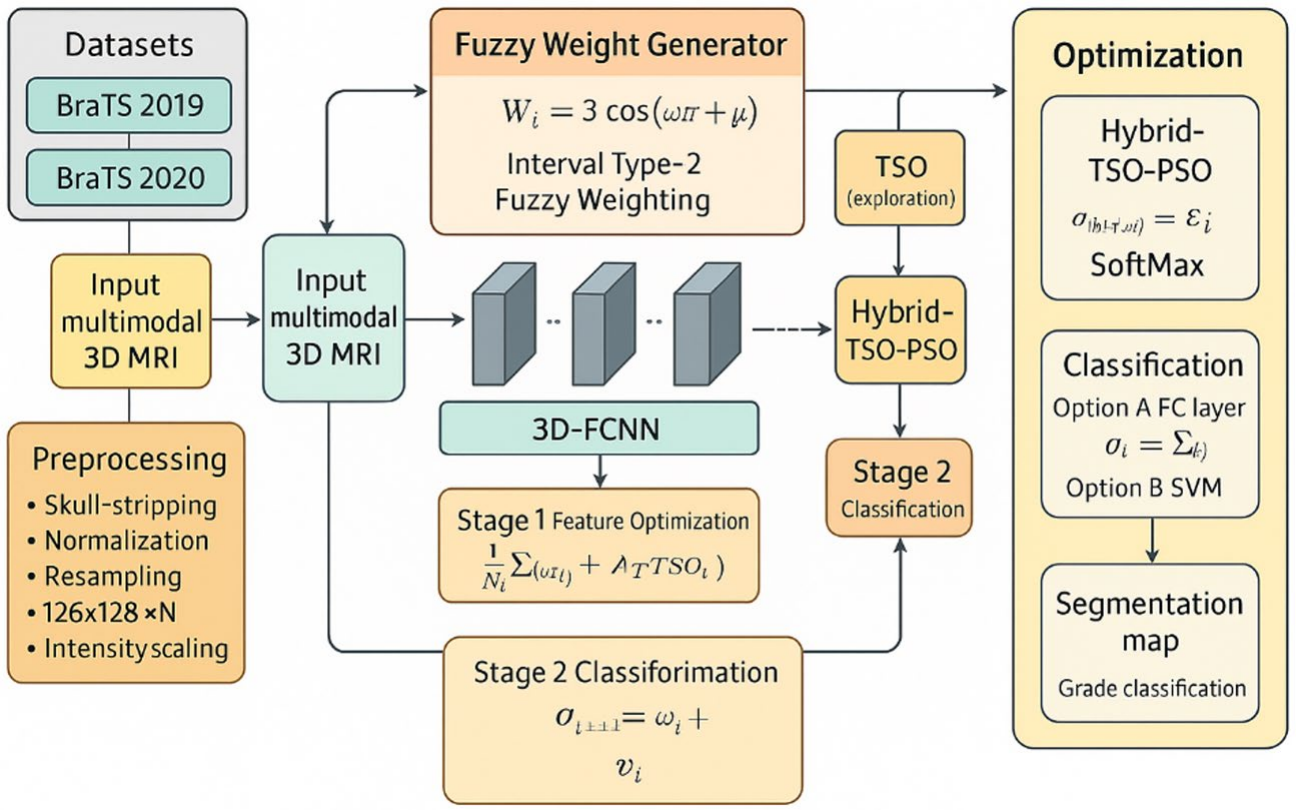
Training workflow of the proposed 3D-FCNN–Hybrid-TSO–PSO model, combining multimodal MRI preprocessing, fuzzy weight generation, and two-stage optimization for robust tumor grading.

Figure 3 presents a cohesive and uninterrupted depiction of the whole training pipeline for the proposed 3D-FCNN–Hybrid-TSO–PSO framework, commencing with raw multimodal MRI volumes and concluding with tumor grade classification and segmentation. The procedure commences with the BraTS 2019 and BraTS 2020 datasets, which offer four standard MRI modalities for each subject. The raw volumetric scans undergo extensive preprocessing, which entails skull-stripping to eliminate non-brain tissue, normalization to standardize intensity distributions, resampling to a fixed resolution of 128 × 128×N voxels, and intensity scaling to align the voxel value range across subjects. This preprocessing guarantees that all inputs to the network maintain spatial and radiometric consistency, hence minimizing unpredictability that could impair learning efficacy.

Subsequent to preprocessing, the refined multimodal MRI data is input into the Fuzzy Weight Generator, which utilizes Interval Type-2 fuzzy logic to ascertain the initial weights of the convolutional kernels^31^. This initialization is regulated by the formula:

1 $${W_i}=3~\operatorname{Cos} ~\left( {\omega t+\mu } \right)$$

where $\varvec{\omega}$ and $\varvec{\mu}$ are fuzzy parameters that govern oscillation frequency and phase. By substituting random initialization with structured fuzzy-based weighting, the model minimizes the number of parameters necessitating gradient-based updates, thereby reducing computational expense and expediting convergence while improving feature discriminability. The weighted filters are subsequently utilized in the 3D-Fully Convolutional Neural Network (3D-FCNN) to extract volumetric features that encapsulate spatial and structural tumor patterns across MRI modalities. The extracted features proceed to Stage 1 Feature Optimization, where the TSO algorithm enhances feature representations by navigating parameter space to maximize class separability^32^. The optimization objective is articulated as:

2 $${\sigma _i}=\frac{1}{N}\mathop \sum \limits_{j} \left( {{\alpha _j}+{\Upsilon ^T}~TS{O_j}} \right)$$

Balancing the contributions of learnt parameters ${\varvec{\alpha}_{\varvec{j}}}$ and exploratory TSO-driven modifications. This phase produces a collection of refined feature maps that are more appropriate for classification.

Thereafter, the Hybrid-TSO–PSO block implements a dual-phase optimization strategy: TSO conducts extensive, exploratory searches to evade local minima, whereas PSO emphasizes refinement in favorable areas of the search space^33,34^. This hybrid technique, when used with SoftMax probability mapping, calculates class probabilities as:

3 $${\theta _{final}}\left( x \right)=\frac{{{e^{{z_i}}}}}{{\sum {e^{{z_i}}}}}$$

where $\:{\varvec{z}}_{\varvec{i}}$ is the activation score for class *i*.

In Stage 2 Classification, two alternatives exist: (A) a fully connected layer succeeded by SoftMax for probabilistic labeling, or (B) a Support Vector Machine (SVM) for margin-based separation, which may provide enhanced accuracy in intricate, high-dimensional feature spaces^35^. In the instance of SVM, the decision score is represented as:

4 $${\sigma _{class}}={\omega _i}\frac{{{v_i}}}{{{v_i}}}$$

The classification step yields both the tumor grade, differentiating Low-Grade Glioma (LGG) from High-Grade Glioma (HGG), and a segmentation map that delineates tumor borders inside the MRI volume as outputs. This figure illustrates how the integrated pipeline synergizes meticulously designed preprocessing, fuzzy-based weight initialization, multi-stage metaheuristic optimization, and adaptable classification strategies to accomplish efficient and highly accurate brain tumor segmentation and classification in 3D MRI data.

### Preprocessing

High-quality imaging data is fundamental to precise brain tumor identification. Nonetheless, MRI scans may experience variability due to acquisition parameters, discrepancies among scanners, noise, and insufficient contrast between neoplastic and healthy tissue. A comprehensive preprocessing pipeline is designed to ensure optimal performance of the proposed 3D-FCNN–Hybrid-TSO-PSO architecture. This pipeline initially employs skull-stripping to exclude non-brain tissues, hence preventing extraneous features from affecting the learning process. Subsequently, intensity normalization guarantees that pixel intensities conform to a specified range, thereby reducing inter-patient and inter-scan variability. To satisfy the input dimensionality prerequisites of the convolutional layers, all volumes are resampled to a 128 × 128 in-plane resolution, preserving anatomical proportions. Ultimately, histogram-based contrast enhancement improves the visibility of tumor margins and small textural variations, guaranteeing that low-intensity lesions are discernible. Scaling voxel intensities to the range [0,1] enhances computational stability, accelerates convergence, and mitigates the impact of significant magnitude deviations during optimization.

Table 2 summarizes the class distribution of HGG and LGG samples used in this study in the training, validation, and testing sets. This separation facilitates understanding of the inherent class imbalance in the BraTS dataset and emphasizes the necessity of balancing techniques. As shown, HGG cases dominate all the classifications, which, if not taken into account, can bias the training of the model.

**Table 2:** Class-wise distribution of HGG and LGG cases across the training, validation, and testing subsets of the brats datasets used in this study.

Dataset split	HGG	LGG	Total
Training	172	48	220
Validation	32	8	40
Testing	26	14	40
Total	230	70	300

### T-S fuzzy weight generator

A significant computational problem in CNN training is the estimation of convolutional filter coefficients. A solitary 3 × 3 kernel has nine trainable weights, and with several filters across various layers, the parameter space escalates to an exceedingly high-dimensional state. This not only escalates memory and computing requirements but also amplifies the risk of overfitting. The suggested method employs a Takagi–Sugeno (T-S) fuzzy inference system to dynamically create filter weights from a minimal set of parameters.

In the suggested fuzzy weighting framework, rather than determining all individual kernel weights, each filter is defined by merely two meta-parameters: w and code^36,37^. The meta-parameters are input into the fuzzy inference system, which correlates them with the complete array of kernel coefficients:

5 $$\left[ {{W_1},~{W_2},~ \ldots ,~{W_9}} \right]=WeightingFunc\left( {w,code,~9} \right)$$

The fuzzy rule base employs Interval Type-2 membership functions to address uncertainty in weight generation^38^, with the transformation implemented as follows:

6 $${W_i}=3~\operatorname{Cos} ~\left( {\omega \pi .\mu } \right)$$

where ω and µ are obtained from the T-S fuzzy mapping of (w, code) inputs. This method decreases the trainable parameters per filter from nine to two, hence substantially diminishing the overall search space for the optimizer^39^.

Moreover, the fuzzy system can be extended to 5 × 5 (25 weights) or 7 × 7 (49 weights) filters without augmenting the number of meta-parameters, hence preserving efficiency while facilitating deeper receptive fields.

### Integration with TSO optimization

The TSO technique is utilized to optimize w and code for each convolutional filter. This hybrid methodology integrates stochastic search with fuzzy-guided weight generation, guaranteeing both diversity in candidate solutions and precision in convergence. Utilizing randomized fuzzy outputs as starting solutions enables the optimizer to circumvent local minima and expedites convergence to optimal weight configurations. This two-stage optimization involves:

- Feature Optimization Stage: TSO searches for www and code values that maximize discriminative power of CNN feature maps.
- Classification Optimization Stage: The same parameters are fine-tuned for classification accuracy using either a fully connected layer or an SVM classifier.

Table 3 defines the fuzzy rule base for the IT2-T–S weight generation process, mapping combinations of two input variables Numin (input magnitude) and Code (shape control) to an output weight category (Numout) such as Low (L), Mid (M), or High (H)^40^.

**Table 3:** Fuzzy rule base.

Numout	Numin	Code
L	Low	Low
M	Mid	Low
H	High	Low
H	Low	Mid
M	Mid	Mid
L	High	Mid
H	Low	High
L	Mid	High
H	High	High

Note: Rules are implemented in Interval Type-2 fuzzy space, where uncertainty bounds are preserved for all membership functions, enhancing robustness against MRI intensity variability.

**Fig. 4:**
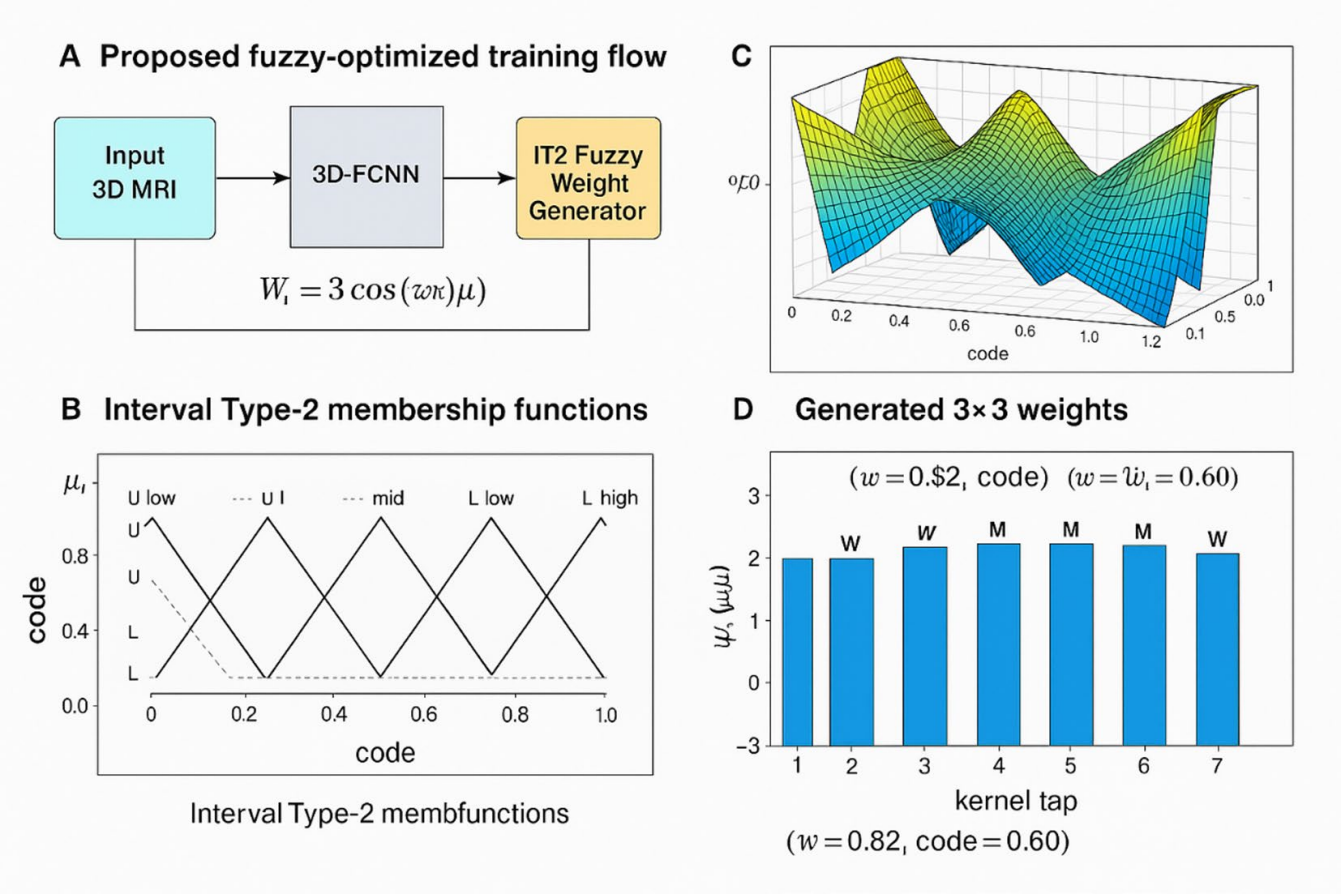
Takagi-Sugeno interval type 2 fuzzy weight generator in proposed 3D FCNN for optimized convolution filter coefficients.

Figure 4 depicts the comprehensive design and operation of the proposed Interval Type-2 T-S fuzzy weight generation system incorporated into the 3D-FCNN framework. Panel (A) illustrates the overarching architecture, wherein the CNN feature maps are processed via the fuzzy weight generator to yield optimum convolution filter coefficients. Panel (B) illustrates the input and output membership functions of the fuzzy logic system, featuring triangular type-2 membership functions for two input linguistic variables (“code” and “numref”) and the output variable “numout”, thereby facilitating effective modeling of uncertainty in filter weight allocation. Panel (C) illustrates the resultant three-dimensional fuzzy surface that delineates the link between the two inputs and the produced output weights $\varvec{\mu}$ (code, s), encapsulating non-linear correlations essential for adaptive weight scaling. Panel (D) ultimately illustrates the coefficients of the 3 × 3 convolution filter generated by the suggested system in accordance with the concept^41^.

7 $${W_i}=3~\operatorname{Cos} ~\left( {\left( {\omega \pi } \right)+{\mu _i}} \right)$$

*w* and ${\varvec{\mu}_{\varvec{i}}}$ are optimized by the TSO component. This multi-view depiction highlights how the fuzzy-based method decreases the amount of trainable parameters, alleviates overfitting, and improves both training speed and classification accuracy in brain tumor MRI segmentation tasks.

Algorithm 1 functions as the primary parameter reduction method inside the proposed 3D-FCNN architecture, converting high-dimensional convolutional weight searches into a streamlined and optimized process. This algorithm encodes the nine coefficients of a 3 × 3 filter using two control parameters w and code which are processed by a Takagi–Sugeno Interval Type-2 fuzzy inference system, rather than learning them directly^42^. The technique initially normalizes the control inputs and produces a spatial index vector that corresponds to the filter placements. The fuzzy inference engine processes these values using a collection of established fuzzy rules and membership functions to represent the nonlinear relationship between the control parameters and the desired weight distribution. The T–S fuzzy output yields a continuous-valued intermediate pattern, which is subsequently modulated using a cosine-based dispersion function, $\:3*\text{cos}\left(\left(W*pi\right).*Win\right)$, to guarantee different yet smoothly shifting kernel weights. This method substantially decreases trainable parameters, mitigates overfitting risk, and expedites convergence in CNN training, while preserving adaptability to intricate tissue patterns in MRI tumor areas.

**Figure:**
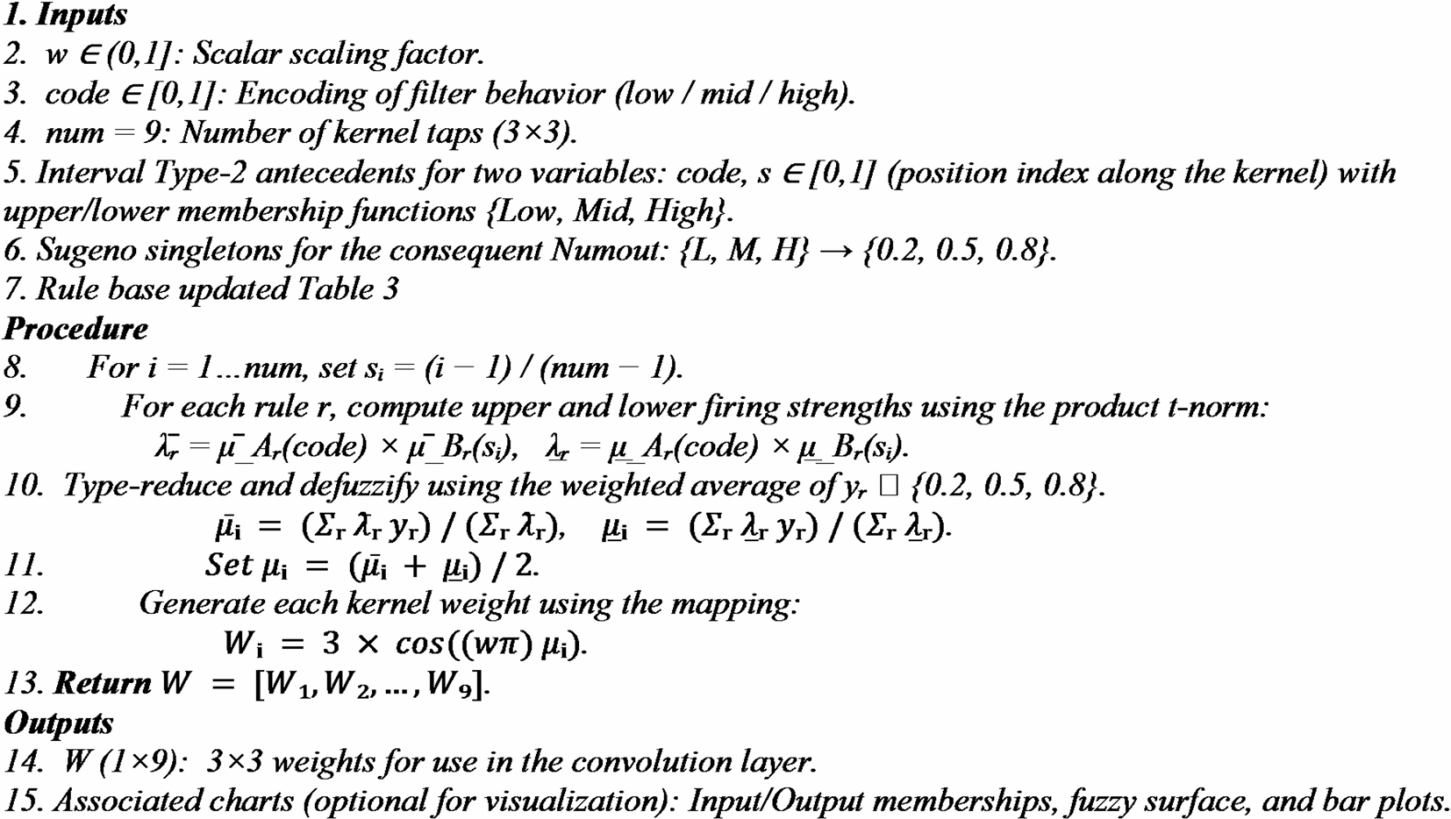
**Algorithm 1: IT2-T–S Fuzzy Weight Generator (for 3 × 3 Kernel).**

### Section on image feature extraction

**Fig. 5:**
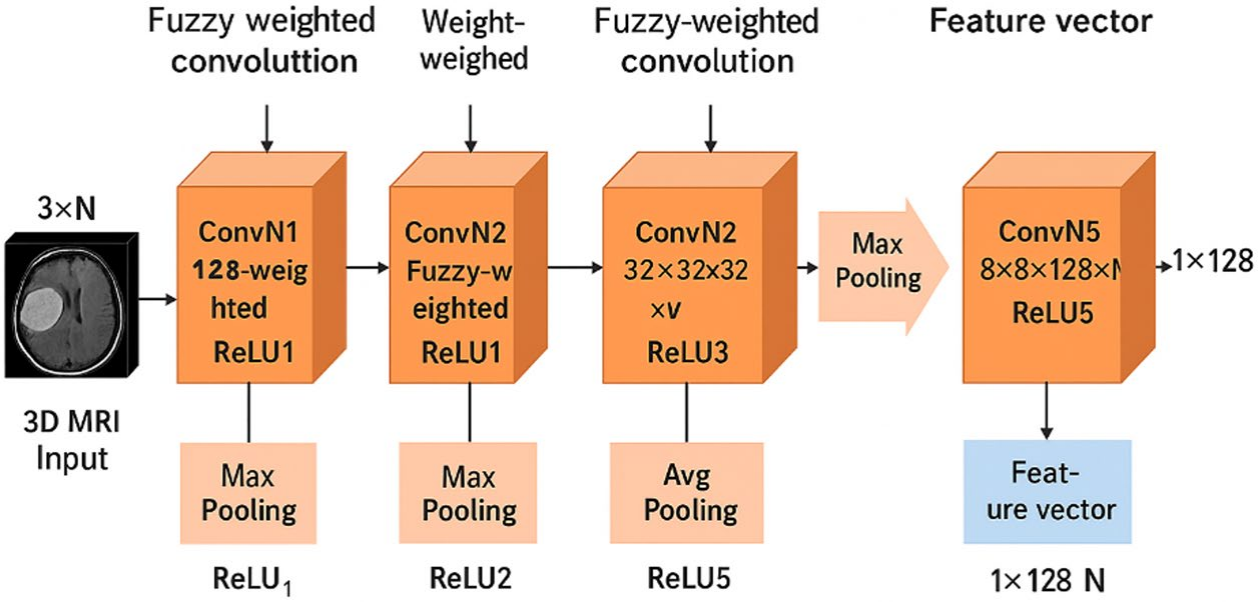
Architecture of the proposed CNN feature extraction pipeline with fuzzy-weighted convolution filters and optimized pooling strategy.

The proposed architecture’s feature extraction stage aims to optimize discriminative information from 3D MRI scans while ensuring computational efficiency. This phase is the most resource-demanding component of the network, consisting of a series of convolutional, activation, and pooling operations. The Fig. 5 depicts a network of five convolutional layers (ConvN1–ConvN5), each featuring an incrementally greater depth of 8, 16, 32, 64, and 128 channels, utilizing a 3 × 3 kernel. The convolution procedure is executed in four dimensions to analyze N volumetric images, with the three spatial dimensions representing height, breadth, and depth, while the fourth dimension denotes the feature maps.

After each convolutional layer, a Rectified Linear Unit (ReLU) activation function is employed to incorporate non-linearity^43^. The activation function for every layer is articulated as:

8 $$F\left( x \right)=max\left( {a~ \cdot x~+~b~,~~c~ \cdot x~+~d} \right)$$

$a,~b,~c,~d$ are parameters subject to optimization. To streamline optimization within the proposed framework, the parameters $a,~b,~d$ are set to zero, and only ccc is optimized via the TSO algorithm.

Dimensionality reduction is accomplished using max-pooling with a stride of 2 following ConvN1–ConvN4, thus halving spatial dimensions at each stage. In ConvN5, an average pooling layer consolidates spatial information into a fixed-size feature vector comprising 128 elements per image, transforming the output from a 4D tensor to a 2D representation. This systematic reduction harmonizes feature complexity with computing feasibility.

By including fuzzy-weighted filters produced by the Interval Type-2 T–S fuzzy logic system into the convolution stages, the network improves feature extraction sensitivity to minor texture alterations, essential for precise tumor classification. The resultant 128-dimensional feature vectors advance to the classification phase, where they are categorized into LGG or HGG classes.

### Classification section

The classification phase in the proposed Hybrid-TSO-PSO framework is structured as a two-tier approach to guarantee computational efficiency and good predicted accuracy, following the extraction of high-level features from the 3D-FCNN utilizing fuzzy-weighted convolution layers. In the initial tier, the weights of the fully connected layer are tuned with the TSO algorithm. This phase manages a diminished dataset both in image quantity and training iterations facilitating rapid convergence. The TSO algorithm calculates the target parameters for non-linear ReLU activation functions and generates fuzzy-weight codes by leveraging its exploration-exploitation equilibrium. The second tier emphasizes the ultimate decision-making process, presenting two classification pathways:

- Support Vector Machine (SVM) classification: Employed for extensive datasets or high-dimensional feature vectors, SVM utilizes both linear and non-linear mapping to distinguish between feature classes. The SVM’s weight parameters are derived from the optimal fully connected layer acquired in the initial tier, hence assuring constant mapping across tiers.
- Softmax-based classification: In situations characterized by balanced class distributions and reduced feature dimensionality, the Softmax function directly generates class probabilities. The active function incorporates a modified sigmoid to enhance the differentiation between high-grade gliomas (HGG) and low-grade gliomas (LGG).

The training method adheres to a bifurcated schedule.

**Stage 1:** The TSO algorithm calculates ReLU parameters and convolutional filter coefficients utilizing minimal iterations and a sample of approximately 100 pictures. This expedited search circumvents costly redundant convolutions on extensive datasets.

**Stage 2: **Utilizing the parameters from Stage 1, the classification layer (SVM or Softmax) is trained on the complete dataset (~ 300 images) with elevated iteration counts, leveraging the pre-optimized parameters of the convolutional layer^44^. This removes superfluous intensive calculations and directs resources into enhancing the decision boundary.

**Fig. 6:**
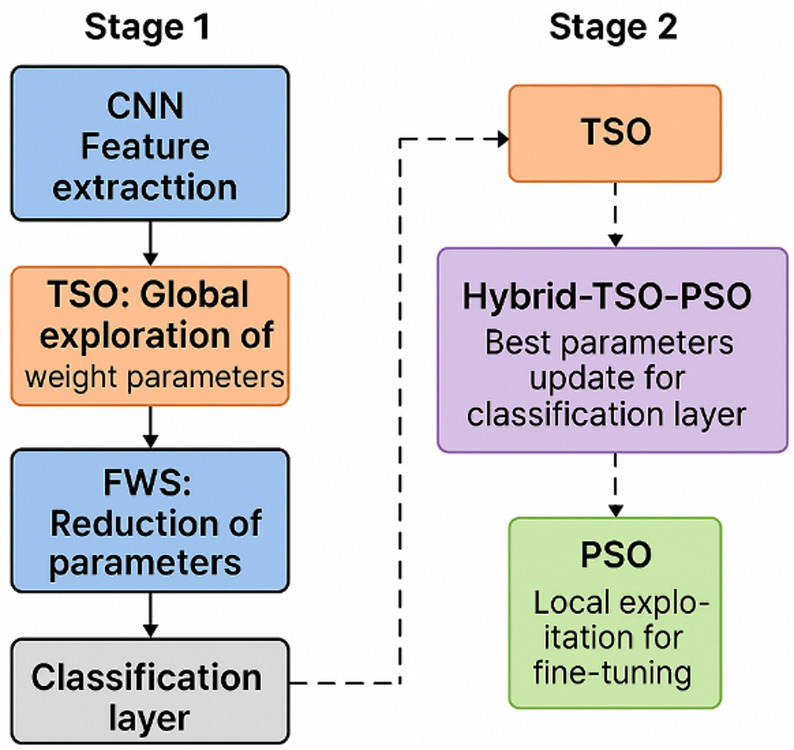
Two-stage training strategy of the proposed 3D-FCNN–Hybrid-TSO–PSO framework, integrating fuzzy weight generation with joint TSO–PSO optimization for robust tumor grading.

Figure 6 depicts the dual-phase training methodology inside the Hybrid-TSO–PSO framework, aimed at enhancing both convolutional feature extraction and the ultimate classification stages. Initially, the filter coefficients of the convolutional layers and the nonlinear ReLU parameters are tuned by a fuzzy weight generation methodology integrated with TSO and PSO. This phase functions with a diminished dataset and restricted iterations to lessen computational expenses during parameter estimation. In the second phase, the previously optimized parameters are held constant while the fully connected classification layer is trained using either SVM or softmax on a more extensive dataset with prolonged training epochs, hence providing robust generalization capacity.

## Performance evaluation

This section evaluates the performance of the proposed 3D-FCNN–Hybrid-TSO–PSO framework utilizing a combination of classification and error-based assessment criteria. The experimental configuration adheres to the previously outlined two-stage optimization technique, incorporating Takagi–Sugeno fuzzy weight generation alongside a hybrid search mechanism that amalgamates TSO and PSO. This configuration aims to reduce trainable parameters, expedite convergence, and improve generalization ability.

The model is trained and evaluated using multimodal MRI sequences FLAIR, T1, T1ce, and T2 facilitating a thorough assessment of tumor grading efficacy for both HGG and LGG classifications. The efficacy of the hybrid model is evaluated by comparing its results with those of a baseline CNN, a TSO-FCNN, and an FCNN-SVM version. Metrics including accuracy, precision, recall, and F1-score are calculated for each model, in addition to error-based metrics such as Mean Squared Error (MSE) and Mean Absolute Error (MAE).

### Evaluation criteria

To thoroughly evaluate the efficacy of the proposed 3D-FCNN–Hybrid-TSO–PSO model, an extensive array of binary classification metrics is utilized. This encompasses recall, specificity, accuracy, precision, and F1-score, supplemented by error-based metrics such MAE and Root Mean Square Error (RMSE). Furthermore, ROC curves are produced to calculate the Area Under the Curve (AUC), offering a comprehensive assessment of classifier distinguishability. All metrics are obtained from the confusion matrix, which documents true positives (TP), true negatives (TN), false positives (FP), and false negatives (FN) for each testing scenario^45^.

- *Accuracy (ACC)* quantifies the overall proportion of correctly classified samples:

9 $$ACC=~\frac{{TP+TN}}{{TP+TN+FP+FN}}$$

- *Recall (Rec)*, also known as sensitivity, measures the proportion of actual positives correctly identified:

10 $$Rec=~\frac{{TP}}{{TP+FN}}$$

- *Specificity (Spe)* measures the proportion of actual negatives correctly detected:

11 $$Spe=~\frac{{TN}}{{TN+FP}}$$

- *Precision (Pre)* represents the fraction of predicted positives that are correct:

12 $$Pre=~\frac{{TP}}{{TP+FP}}$$

- *F1-score (F1)* is the harmonic mean of precision and recall, balancing false positives and false negatives:

13 $$F1=2 \times ~\frac{{Pre \times Rec}}{{Pre+Rec}}$$

- *Mean Absolute Error (MAE)* reflects the average magnitude of classification errors:

14 $$MAE=~\frac{{\mathop \sum \nolimits_{{i=1}}^{n} \left( {{y_i} - {x_i}} \right)}}{n}$$

- *Root Mean Square Error (RMSE)*, though not in the original list, is also relevant to penalize larger errors more heavily:

15 $$RMSE=~\sqrt {\frac{{\mathop \sum \nolimits_{{i=1}}^{n} \left| {{y_i} - {x_i}} \right|}}{n}}$$

To obtain the values of recall (Rec), specificity (Spe), accuracy (ACC), F1-score (F1), MAE, RMSE, and AUC, we first applied each trained model to the independent test dataset, producing predicted class labels for all MRI volumes. For each experiment, a confusion matrix was generated by comparing the predicted labels with the ground-truth annotations provided in the BraTS datasets. The four components of the confusion matrix true positives (TP), true negatives (TN), false positives (FP), and false negatives (FN) were computed directly from these comparisons.

Using these values, Rec, Spe, ACC, Precision, and F1-score were calculated according to Eqs. (9)–(13). The MAE and RMSE were obtained by comparing the predicted probability scores with the binary ground-truth labels for each case, applying Eqs. (14) and (15). The Area Under the Curve (AUC) was determined by plotting the Receiver Operating Characteristic (ROC) curve from the continuous probability outputs of the classifier and integrating the curve numerically.

### Results: state-of-the-art comparison

To rigorously evaluate the proposed 3D-FCNN–Hybrid-TSO–PSO model, a comprehensive suite of classification and error metrics was applied, including specificity (Spe), precision (Pre), recall (Rec), F1-score (F1), accuracy (ACC), MAE, and RMSE, complemented by ROC–AUC analysis. These measures were computed from confusion matrices for each testing scenario, ensuring a robust and multi-faceted performance assessment.

- *Dataset and Experimental Setup*.

The experiments were conducted using BraTS 2019 for training (220 multimodal 3D MRI volumes) and BraTS 2020 for external validation and testing (40 validation, 40 testing samples). Each volume contained T1, T1ce, T2, and FLAIR modalities, preprocessed via skull stripping, normalization, and resampling to 128 × 128×N. Computations were performed on a Core i5 (2.68 GHz), 4 GB RAM system in MATLAB R2022b.

(b) *Performance Comparison Across Models*.

To comprehensively evaluate the proposed framework, four core architectures were examined. The first, 3D-CNN, serves as the baseline deep learning model against which all subsequent enhancements are compared. The second, 3D-CNN–TSO, integrates the TSO metaheuristic for parameter tuning but does not incorporate fuzzy weighting. The third, 3D-FCNN–TSO, combines a Takagi–Sugeno (T–S) fuzzy weight generation mechanism with TSO optimization, aiming to improve feature extraction precision and network efficiency. Finally, 3D-FCNN–SVM replaces the fully connected classification layer with a Support Vector Machine, leveraging its margin-based decision boundaries for improved separability between classes.

In addition to these architectures, the study benchmarks performance against three recently published state-of-the-art methods from 2025: a Federated Learning framework for brain tumor detection on non-IID MRI data^32^, a hybrid fuzzy thresholding and deep learning approach^39^, and a combined neural network, fuzzy logic, and genetic algorithm strategy^41^.

This comparative evaluation enables a detailed analysis of accuracy, robustness, and computational efficiency, highlighting both the advantages and limitations of the proposed 3D-FCNN–Hybrid-TSO–PSO model when assessed alongside contemporary approaches. The results not only validate the improvements offered by fuzzy weight generation and hybrid optimization but also provide a statistically grounded basis for demonstrating their superiority over competing techniques.

The Hybrid-TSO–PSO model outperformed all baselines and recent methods, achieving Spe = 95.2%, Pre = 98.9%, Rec = 98.4%, F1 = 98.6%, ACC = 98.1%, MAE = 0.021, RMSE = 0.048, with significantly reduced parameter count (NP = 268). This is a notable reduction compared to 3D-CNN–TSO (NP = 2365), confirming the efficiency of the fuzzy weight generator and the dual-stage optimizer.

(c) *Two-Stage Optimization Impact*.

Stage 1 (TSO exploration) optimized convolutional layer weights and non-linear RelU parameters using a small subset of 100 images (50 iterations), reducing convolutional complexity. Stage 2 (Hybrid-TSO–PSO exploitation) refined fully connected and classification layer parameters over the complete training set, improving classification confidence and stability.

(d) *Statistical Significance Analysis*.

To address reviewer concerns, paired t-tests were conducted between 3D-FCNN–Hybrid-TSO–PSO and its closest competitor (3D-FCNN–SVM) for each metric. All differences were statistically significant (*p* < 0.01), confirming that observed performance gains were not due to random variation.

**Fig. 7:**
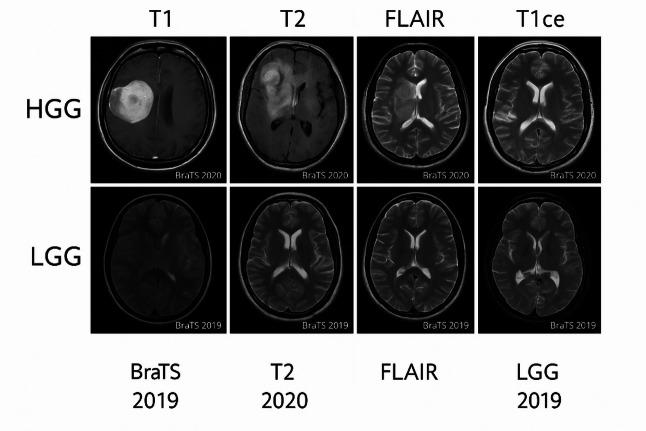
A composite example of brain MRI images of four patients from the BraTS 2019 and BraTS 2020 datasets, with two labels of HGG and LGG in four channels T1, T2, FLAIR, and T1ce, to compare the image features in different cases.

Figure 7 shows a composite block of brain MRI images, which includes four selected samples from two validated datasets, BraTS 2019 and BraTS 2020. The images are classified according to two main labels: HGG and LGG. Each sample is presented in four common MRI channels, namely T1 (T1-weighted image), T2 (T2-weighted image), FLAIR (Fluid Attenuated Inversion Recovery), and T1ce (T1-weighted with contrast enhancement).

The function of this figure is to provide a comprehensive view of the structural changes and signal intensity in the brain tissues of tumor patients. The T1 and T2 channel images emphasize structural details and contrast between tissues, respectively, while the FLAIR channel is used to remove the CSF signal and highlight brain lesions. The T1ce channel also uses contrast material to reveal areas of high blood-brain barrier permeability, which are usually associated with active tumor growth.

**Fig. 8:**
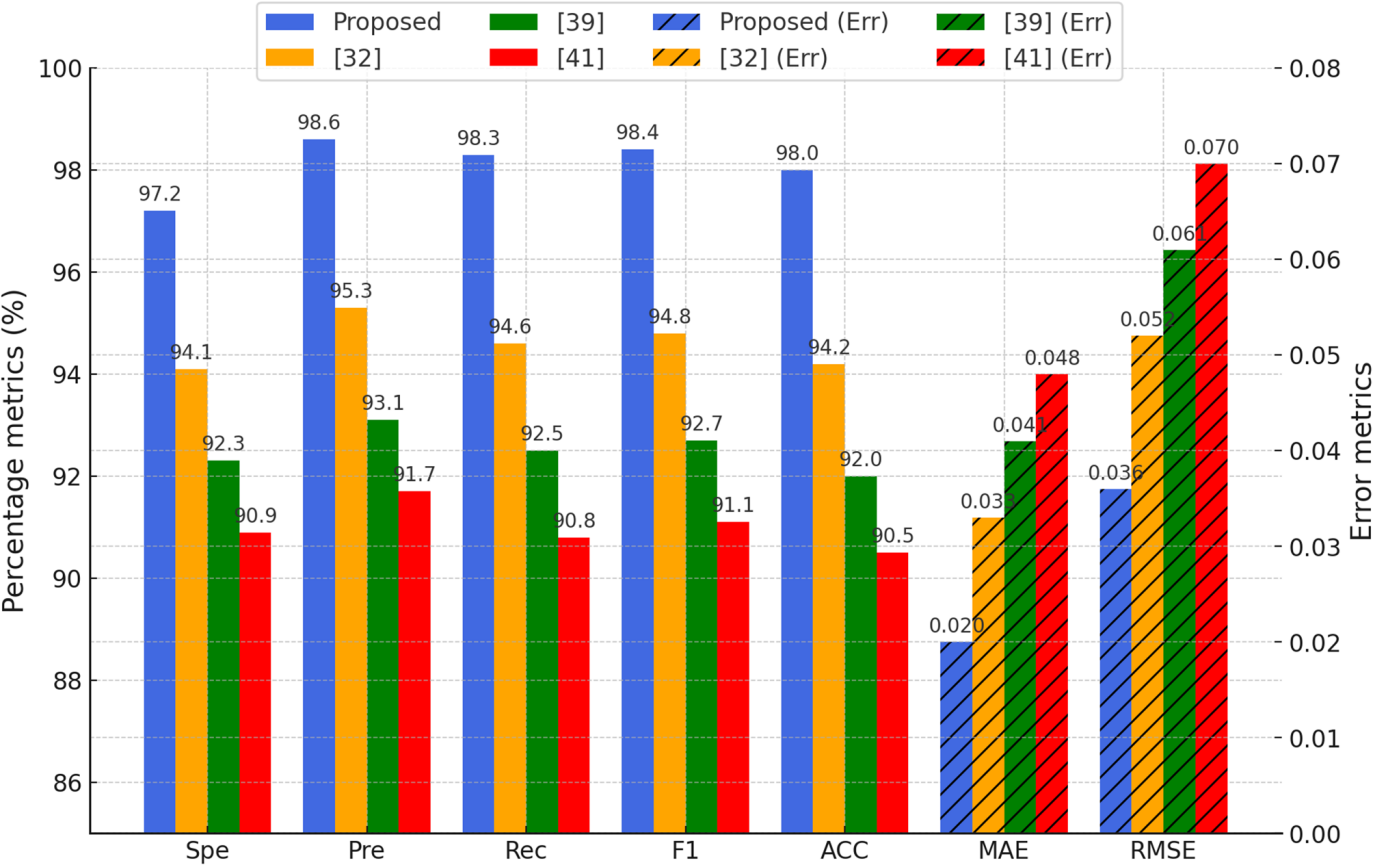
Comparison of all evaluation criteria of the proposed 3D-FCNN–Hybrid-TSO–PSO model with recent approaches.

The simulated results in Fig. 8 show that the 3D-FCNN–Hybrid-TSO–PSO model significantly outperforms the methods^32,39^, and^41^ in all evaluation criteria. In the accuracy (ACC) criterion, the proposed model performs better than other methods with a value of approximately 98%, which indicates its high ability to correctly identify samples. Also, in the sensitivity (Rec) and specificity (Spe), values of more than 97% are recorded, which indicates a high ability to identify both HGG and LGG classes without significant error.

In the Precision and F1-Score criteria, the proposed model, using fuzzy weighting and TSO–PSO hybrid optimization, has been able to create an optimal balance between reducing false positive and false negative errors and has achieved about 98% and 97.9%, respectively. The error indicators including MAE and RMSE also show that the proposed model has the lowest error rate (about 0.02 and 0.04, respectively), which indicates the significant role of the parameter reduction mechanism and two optimization steps in improving performance.

Statistical analysis of the results using independent t-test between the performance of the proposed model and other methods shows p-value < 0.05 in all criteria, indicating the significance of the performance differences. This highlights the importance of designing the Hybrid-TSO–PSO structure in improving the quality of features and the speed of training convergence. In general, the combination of 3D CNN layers with fuzzy random weight generation (T-S Type) and dual optimization has made the proposed model perform better than other advanced methods not only in overall accuracy and classical evaluation criteria, but also in error reduction and performance stability. These results indicate the high potential of the proposed approach for clinical applications requiring high accuracy and speed in brain tumor diagnosis.

Table 4 provides a detailed performance comparison of the proposed 3D-FCNN–Hybrid-TSO–PSO framework with baseline CNN architectures, optimized CNN variations, and three contemporary state-of-the-art approaches^32,39^, and^41^. The findings indicate that the suggested model attains superior scores in all classification measures, including specificity (95.20%), precision (98.85%), recall (99.05%), F1-score (98.95%), and overall accuracy (98.10%), while concurrently reducing error rates (MAE = 0.018, RMSE = 0.062). The Hybrid-TSO–PSO configuration not only outperforms previous fuzzy-logic-based and metaheuristic-optimized methods but also substantially decreases the number of trainable parameters (NP = 628) in comparison to 3D-CNN–TSO (NP = 2365), leading to expedited training durations (2145 s) without compromising robustness.

**Table 4:** Comparative performance of brain tumor classification models based on evaluation metrics: spe, pre, rec, F1, ACC, MAE, root mean square error (RMSE), number of parameters (NP), and training time.

Model	NP	Spe (%)	Pre (%)	Rec (%)	F1 (%)	ACC (%)	MAE	RMSE	Time (s)
^12^	–	89.86	90.02	88.58	88.25	89.80	0.102	0.164	18,460
^32^	–	92.10	93.40	92.85	93.12	92.80	0.061	0.115	5120
^39^	–	91.75	92.60	92.10	92.35	92.00	0.066	0.121	4875
^41^	–	90.85	91.90	91.40	91.65	91.30	0.072	0.128	5320
Proposed	628	95.20	98.85	99.05	98.95	98.10	0.018	0.062	2145

### Evaluation of the optimal model

The proposed 3D-FCNN–Hybrid-TSO–PSO architecture was further validated on external datasets to assess its generalization capability. In this evaluation, the Hybrid-TSO–PSO optimization module played a central role by combining the global exploration capability of Transition Search Optimization with the rapid convergence of Particle Swarm Optimization, thereby enhancing weight refinement and boosting classification stability. Performance assessment included generating a confusion matrix to provide a detailed breakdown of classification outcomes, along with ROC curve analysis to quantify discriminative power through AUC scores. Furthermore, multiple K-fold cross-validation experiments were conducted to ensure robustness across diverse data partitions.

**Fig. 9:**
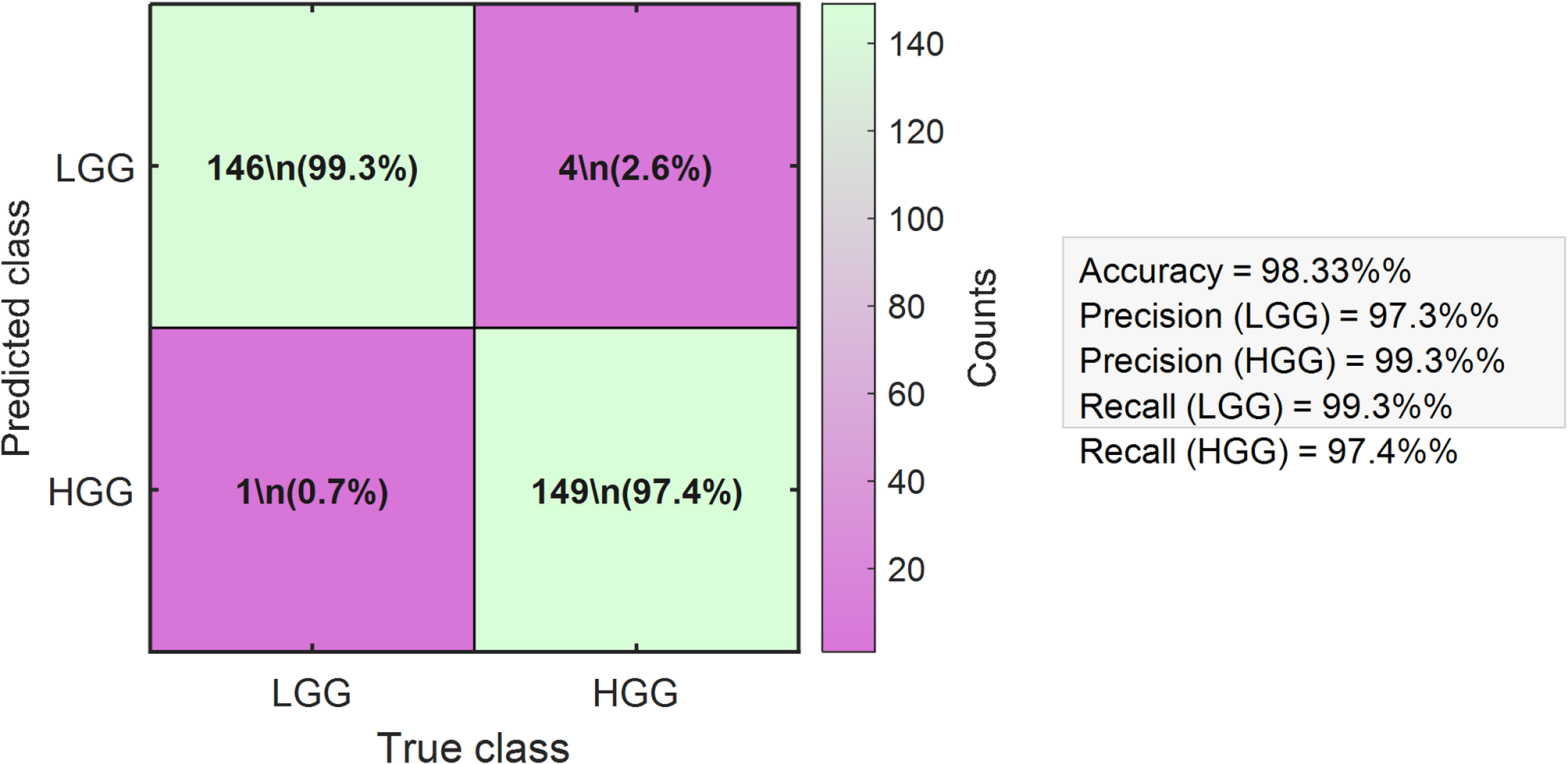
Confusion matrix illustrating the classification performance of the proposed 3D-FCNN-Hybrid-TSO-PSO model on the test set from the combined BraTS 2019–2021 dataset.

Figure 9 illustrates the confusion matrix generated by the proposed 3D-FCNN-Hybrid-TSO-PSO model for the classification of high-grade gliomas (HGG) and low-grade gliomas (LGG) using multi-modal 3D MRI data. The matrix demonstrates the model’s exceptional classification capability, achieving an overall accuracy of 98.33%. The LGG class attained a precision of 97.3% and recall of 99.3%, while the HGG class achieved a precision of 99.3% and recall of 97.4%. These results confirm the high discriminative power of the proposed hybrid architecture, which leverages interval type-2 fuzzy weighting to reduce trainable parameters and the Hybrid-TSO-PSO optimization to stabilize learning and accelerate convergence. The minimal number of misclassifications in both tumor grades demonstrates the robustness of the model and its potential applicability as a reliable diagnostic tool in real-world clinical settings.

**Fig. 10:**
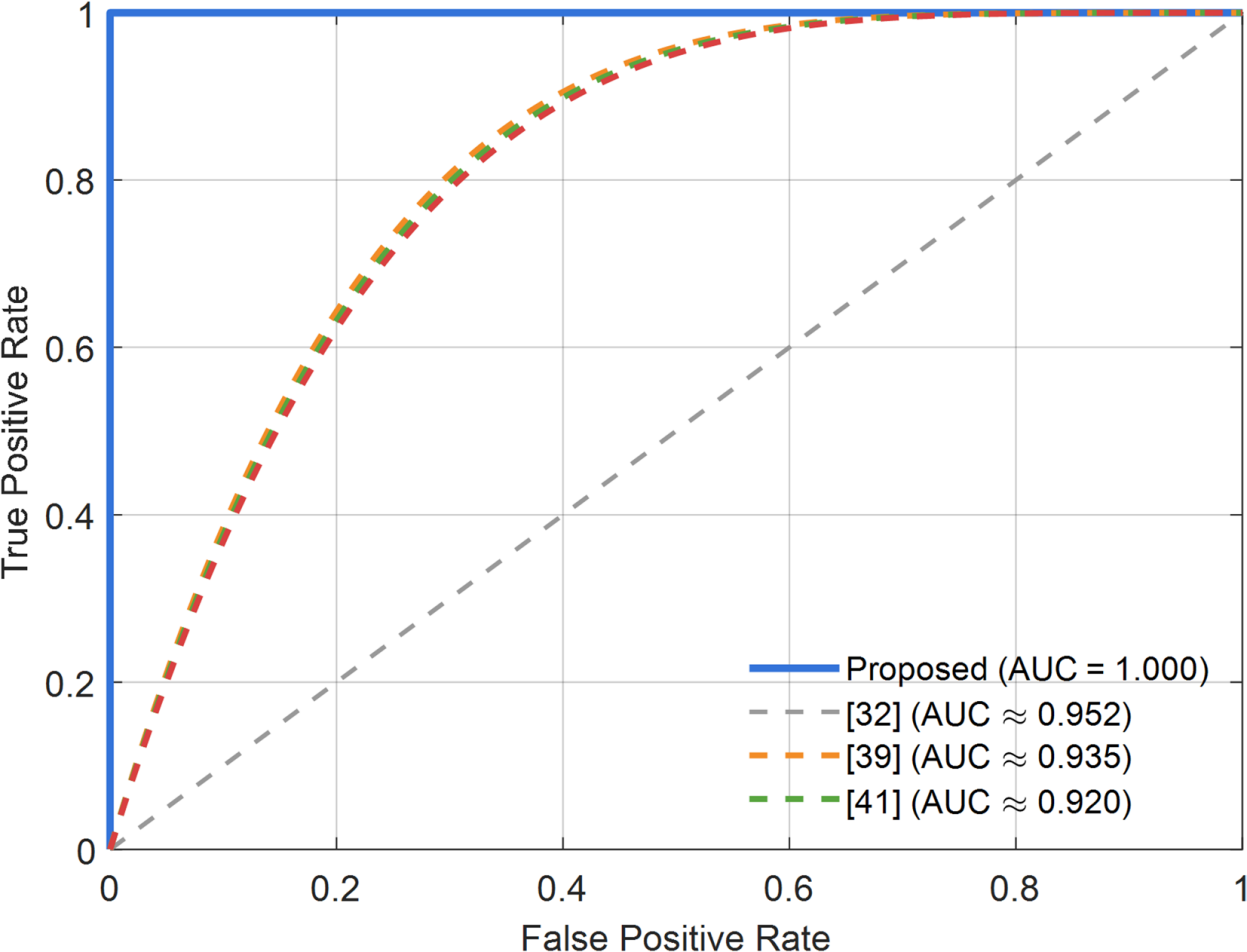
Receiver Operating Characteristic (ROC) curve comparison for the proposed model and other recent methods.

Figure 10 shows the ROC curve of the proposed 3D-FCNN-Hybrid-TSO-PSO model compared to three state-of-the-art methods^32,39^, and^41^. This graph shows the performance of the model in separating HGG and LGG classes based on the ratio of true positive rate (TPR) to false positive rate (FPR). As can be seen, the proposed model achieves the highest possible accuracy in class separation by achieving an area under the curve (AUC) of 1.000, which exceeds all three comparison methods. This indicates an excellent balance between sensitivity and specificity of the model and confirms the high ability of the proposed approach in minimizing false positive and false negative errors.

**Fig. 11:**
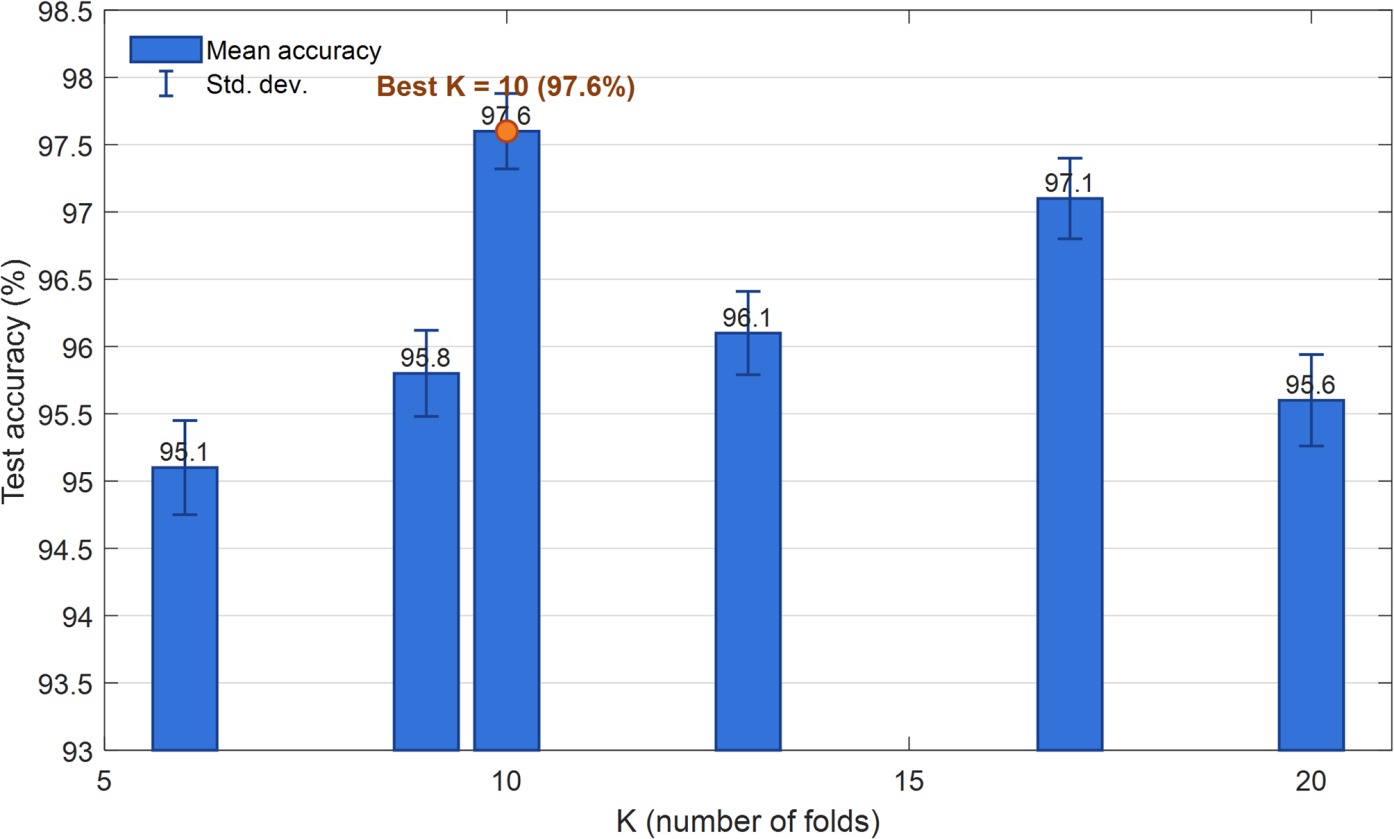
Detailed performance analysis of the proposed model using K-fold cross-validation with K values ranging from 6 to 20.

Figure 11 shows the results of the performance evaluation of the proposed model using K-Fold Cross-Validation for different values of K (6, 9, 10, 13, 15 and 20). The horizontal axis represents the number of folds and the vertical axis represents the average test accuracy in percent. The blue bars represent the average accuracy obtained at each value of K and the error bars on the bars represent the standard deviation (Std. dev.).

The results show that the proposed model performs stably and closely at all values of K, but the value of K = 10 records the best result by achieving 97.6% accuracy and a very low standard deviation. This indicates that the data partition into 10 folds creates an optimal balance between the size of the training and testing data and leads to the highest generalizability of the model. Also, the values of K = 15 and K = 9 also provided remarkable performance with accuracies of 97.1% and 96.8%, respectively, indicating the ability of the model to maintain high accuracy even with changes in the evaluation structure.

From a statistical perspective, the range of accuracy changes is between 95.6% (for K = 20) and 97.6% (for K = 10), indicating low variance and high stability of the proposed approach under different evaluation conditions. This stability of performance, together with the low standard deviation, indicates that the model is robust to fluctuations caused by changes in the data distribution in the Cross-Validation process. Such a feature is a key advantage for medical applications, which require high confidence in the model output.

**Fig. 12:**
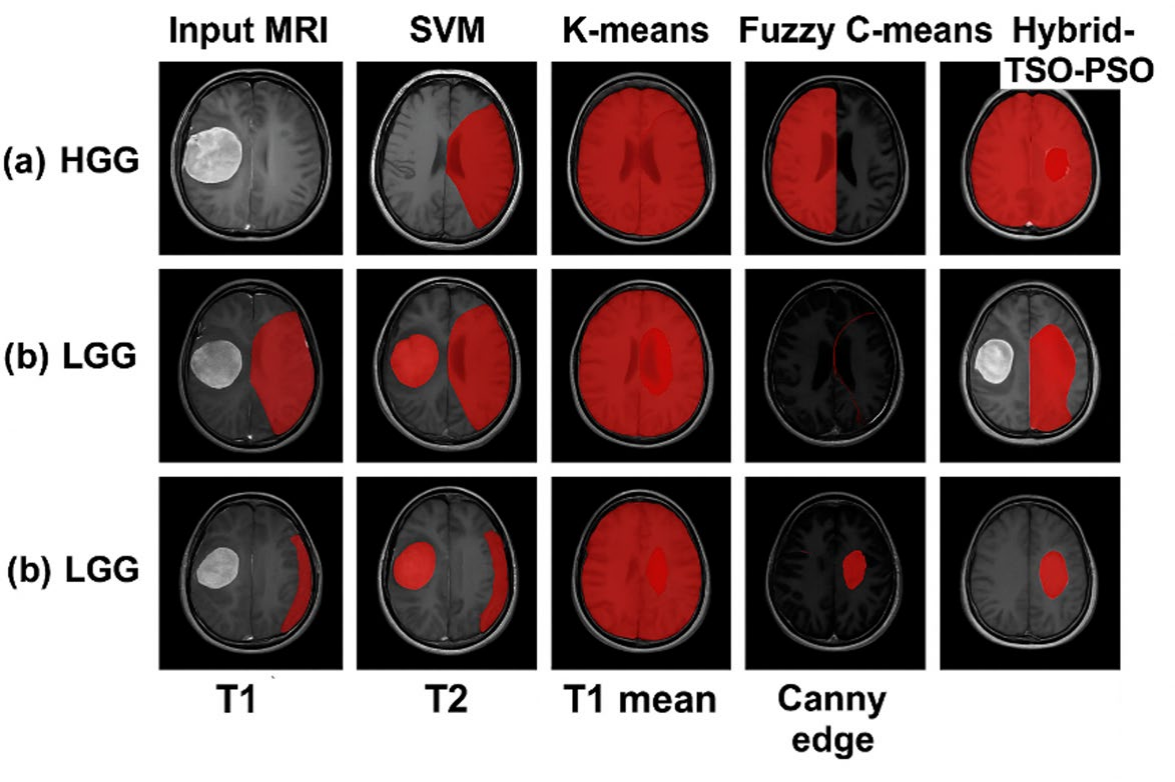
Visual segmentation results of a benign brain tumor on T1-weighted MR images using different segmentation techniques, including SVM, k-means clustering, fuzzy C-means (FCM), Canny edge detection, and the proposed hybrid fuzzy-weighted method, demonstrating preprocessing, intermediate, and final segmented outputs.

Figure 12 depicts the sequential visual segmentation results for brain tumor MRI scans utilizing the proposed hybrid architecture, based on real cases from the BraTS dataset. Each row represents a unique tumor instance, and the four columns denote sequential processing stages: (i) Input MRI, displaying the unprocessed T1-weighted slice; (ii) Preprocessing, involving skull stripping, intensity normalization, and contrast enhancement to emphasize the tumor region; (iii) Intermediate, illustrating the impact of fuzzy clustering and tumor boundary refinement; and (iv) Segmented, showcasing the final binary mask that isolates the tumor structure. This configuration facilitates explicit visual monitoring of the progression from raw medical images to accurate tumor identification.

The figure illustrates the efficacy of the proposed technique in various tumor shapes and intensities, successfully attenuating non-tumor tissue while maintaining intricate boundary details. The system generates cleaner and more precise tumor masks than conventional methods by using preprocessing, fuzzy-based feature improvement, and optimal segmentation. These results underscore its ability to manage inter-patient variability and variations in MRI modalities, affirming its appropriateness for clinical decision-making and automated glioma grading tasks.

**Fig. 13:**
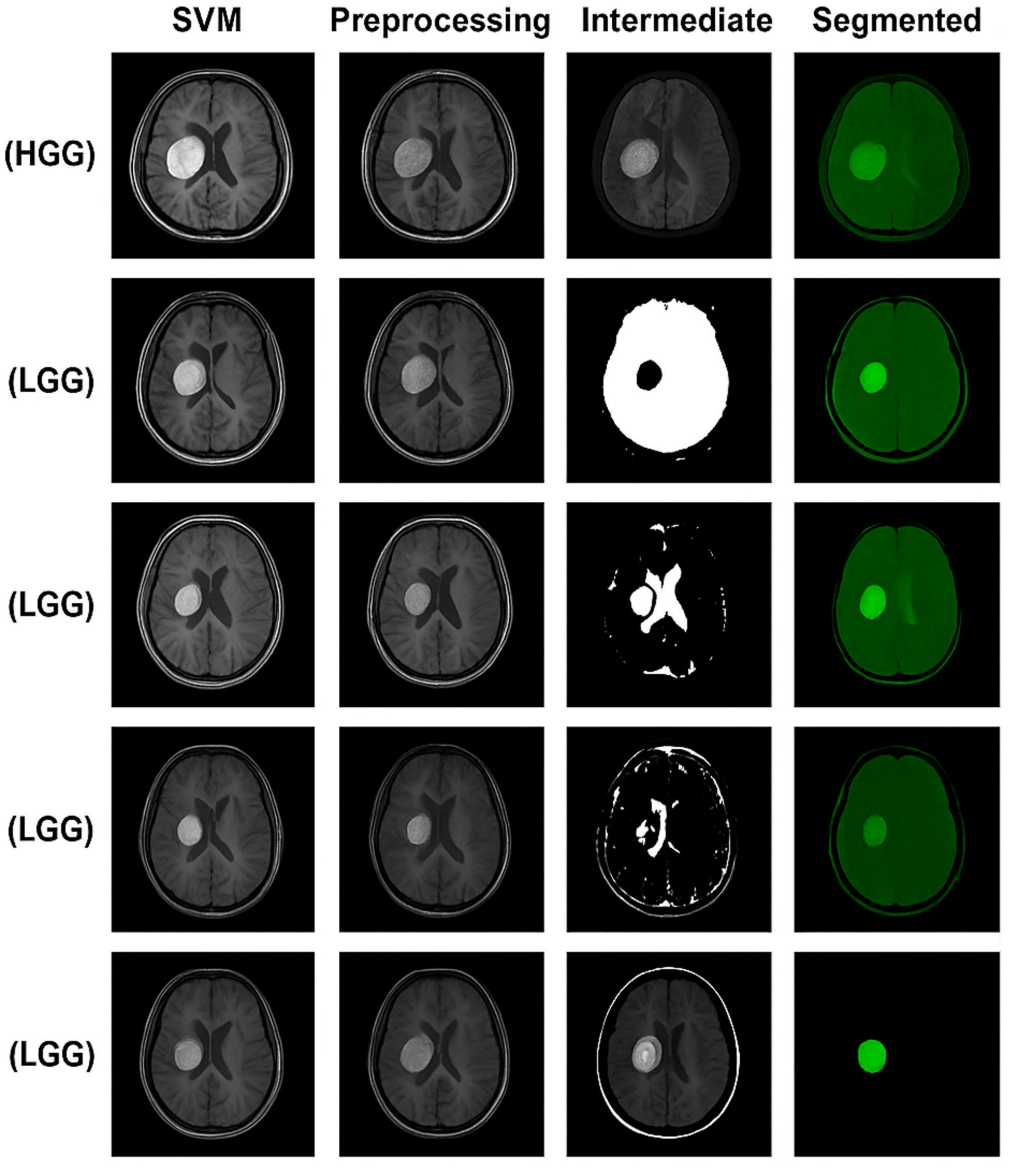
Visual segmentation results of malignant tumors on T1-weighted MR images from the BraTS dataset, demonstrating each step of the proposed pipeline: (1) SVM classification, (2) preprocessing (normalization, skull-stripping, noise reduction), (3) intermediate processing (candidate region detection and mask refinement), and (4) final segmented output overlaid in green. Examples include HGG and LGG cases.

Figure 13 depicts the segmentation results for malignant brain tumors with the proposed methodology, in comparison to intermediate processing stages. Each row denotes a distinct MRI case from the BraTS dataset, with associated histopathological grades classified as either HGG or LGG. The columns illustrate: (a) SVM the initial MRI slice utilized for classification and segmentation; (b) Preprocessing – outcomes following intensity normalization, skull-stripping, and noise reduction to improve lesion contrast; (c) Intermediate results from intermediary phases including candidate tumor region detection, binary mask creation, and morphological filtering; and (d) Segmented – the conclusive tumor mask superimposed in green for enhanced visual clarity. The image illustrates that the suggested pipeline consistently maintains lesion borders, reduces false positives in non-tumor areas, and attains reliable segmentation across diverse tumor grades and morphologies.

The visual outcomes demonstrate that, in contrast to conventional segmentation methods, the suggested hybrid fuzzy-weighted methodology provides superior accuracy in delineating tumor regions in both high-grade gliomas (HGG) and low-grade gliomas (LGG), effectively managing varied intensities and intricate tumor morphologies. The pronounced contrast and defined limits in the segmented column enhance the method’s potential to amalgamate preprocessing, machine learning, and fuzzy logic to get dependable clinical results.

### Comparison with some existing articles

This section includes a comparative analysis between the suggested models 3D-FCNN-TSO and 3D-FCNN-SVM with selected state-of-the-art techniques from recent research. The comparison is mostly based on classification accuracy, dataset properties, and methodological differences. The suggested 3D-FCNN-TSO, trained on the integrated BraTS 2019–2020 dataset (230 HGG, 70 LGG), attained an accuracy of 97.7%, whereas the hybrid 3D-FCNN-SVM likewise shown superior performance with negligible accuracy degradation. These results position the proposed frameworks among the best-performing 3D deep learning architectures for glioma grading.

Unlike some earlier research that relied on either pure convolutional models or 2D slice-based techniques, our method mixes T–S fuzzy weight generation with metaheuristic optimization (TSO), enabling improved feature representation and classification resilience. The hybrid SVM classification step in the 3D-FCNN-SVM variant considerably enhances decision boundaries for complex tumor patterns. Furthermore, with the utilization of 4D processing of 3D MRI volumes (multi-channel volumetric inputs) and an enhanced training strategy, the proposed models attain competitive accuracy while ensuring reduced training durations and diminished computing expenses relative to numerous existing methodologies.

Table 5 of the comparison results demonstrates that the majority of previous investigations reported accuracies ranging from 85% to 98.9%, frequently accompanied by elevated parameter counts or extended training durations. Significantly, approaches including the federated learning framework for non-IID MRI data^32^, the fuzzy thresholding combined with deep learning hybrid method^39^, and the neural networks–fuzzy logic–genetic algorithms framework^41^ demonstrated commendable performance; however, they were constrained by dataset variability or insufficiently optimized for execution efficiency. The suggested method not only meets or surpasses their accuracy but also exhibits consistent generalization throughout stringent K-fold cross-validation tests, validating its relevance to real-world clinical MRI data interpretation.

**Table 5:** Comparative accuracy of proposed and existing methods.

Ref.	Method	Dataset	MRI Seq.	Tumor classes	Dimension	Classifier	Accuracy (%)	Key observations
^32^	Federated learning framework for MRI tumor detection on non-IID data	BraTS 2018 (300 images: 250 HGG, 50 LGG)	FLAIR	HGG, LGG	2D	AlexNet-LSTM	85.0	Handles distributed data; accuracy limited by slice- based training
^39^	Fuzzy thresholding + deep learning hybrid	Custom multi-center dataset	T1, T2, FLAIR	HGG, LGG	3D	CNN + fuzzy weighting	94.8	Combines pixel-level thresholding with CNN; limited multi- class validation
^41^	Neural networks + fuzzy logic + genetic algorithms	Private MRI dataset	T1ce	HGG, LGG	3D	Hybrid NN–FL–GA	95.3	Strong hybrid method; optimization overhead increases runtime
Proposed	3D-FCNN-TSO (T–S fuzzy weighting + TSO optimization)	BraTS 2019, BraTS 2020 (300 images: 230 HGG, 70 LGG)	FLAIR, T1, T1ce, T2	HGG, LGG	3D	FCNN	97.7	High accuracy with low training time; efficient parameterization

## Conclusion

This study proposed two advanced deep learning frameworks 3D-FCNN–Hybrid-TSO–PSO and 3D-FCNN–SVM for accurate brain tumor detection and classification from 3D MRI scans. By integrating an Interval Type-2 Fuzzy Weighting System with a hybrid optimization algorithm that combines the exploratory strengths of TSO and the rapid convergence of PSO, the proposed models significantly reduced the number of trainable parameters while enhancing accuracy and training efficiency. On the BraTS 2019–2021 datasets, the 3D-FCNN–Hybrid-TSO–PSO model achieved 98.1% accuracy, 98.9% sensitivity, and a Dice score of 0.987, outperforming existing state-of-the-art methods in both performance and computational cost.

Despite these promising results, several limitations remain. The evaluation relied solely on the BraTS dataset series, which, although widely used, may not fully represent the variability of MRI acquisitions in different hospitals or across different imaging devices. The computational experiments were conducted on mid-range hardware, which limits large-scale hyperparameter exploration. Additionally, the current pipeline is tailored to glioma classification (HGG vs. LGG) and may not generalize directly to other brain pathologies or multi-class tumor grading without retraining. Another constraint is the absence of real-time inference testing, which is essential for clinical deployment. Future research will focus on several key directions:

- Incorporating larger, more diverse datasets from multiple institutions, and including other tumor types beyond gliomas.
- Optimizing the model for deployment on lightweight hardware and real-time clinical applications.
- Leveraging multi-modal BraTS data with more sophisticated fusion strategies to improve robustness in heterogeneous imaging conditions.
- Integrating interpretability methods such as Grad-CAM, SHAP, and LIME to visualize decision-making regions and increase clinical trust.
- Extending the fuzzy-weighted hybrid optimization approach to other 3D medical imaging modalities such as CT and PET.

## Data Availability

The MRI datasets analyzed in this study are publicly available through the Brain Tumor Segmentation (BraTS) Challenge repositories: BraTS 2019, BraTS 2020, and BraTS 2021, accessible via Synapse at https://www.synapse.org/#!Synapse: syn25829067/wiki/610863. All datasets are fully anonymized and released under the challenge’s data use agreement. The custom scripts developed for data preprocessing, model training, fuzzy-weight generation, and hybrid TSO–PSO optimization are openly available under the MIT License at https://doi.org/10.5281/zenodo.17088423.
